# Effect of genotypes on macronutrients and antioxidant capacity of chicken breast meat

**DOI:** 10.5713/ajas.19.0736

**Published:** 2020-11-01

**Authors:** Phatthawin Lengkidworraphiphat, Rawiwan Wongpoomchai, Sirinya Taya, Sanchai Jaturasitha

**Affiliations:** 1 Department of Animal and Aquatic Sciences, Faculty of Agriculture, Chiang Mai University, Chiang Mai 50200, Thailand; 2 Department of Biochemistry, Faculty of Medicine, Chiang Mai University, Chiang Mai 50200, Thailand; 3 Science and Technology Research Institute, Chiang Mai University, Chiang Mai 50200, Thailand

**Keywords:** Chicken Breast, Antioxidant, Carnosine, Anserine

## Abstract

**Objective:**

The increasing consumer awareness of food, which can provide health benefits and potentially aid disease prevention, has become the driving force of the functional food market. Accordingly, the aim of this study was to investigate the effects of chicken genotype on the macronutrient content, bioactive peptide content, and antioxidant capacity within different breast meat.

**Methods:**

In this experiment, three genotypes of chicken, Thai indigenous, black-boned, and broiler (control), were reared with commercial feed under the same conditions. Thirty chickens were slaughtered at typical market age and the breasts were separated from the carcass to determine macronutrient content using the AOAC method. The antioxidant capacities of the chicken breasts were evaluated by *in vitro* antioxidant assays and the protein pattern was investigated using gel electrophoresis. Carnosine and anserine, which have antioxidant properties in animal tissue, were determined using high performance liquid chromatography.

**Results:**

The results showed that breast meat from Thai indigenous chickens had a greater macronutrient content and higher antioxidant capacity compared with the other genotypes (p<0.05). The protein pattern was similar between genotypes, however Thai indigenous chickens had the greatest myosin and actin content (p<0.05). In addition, carnosine and anserine values were greatest in the black-boned and Thai indigenous chickens compared with the broiler genotype (p<0.05).

**Conclusion:**

Thai indigenous chicken breast meat may be classified as a functional food as it has good nutritional value and is rich in antioxidant peptides.

## INTRODUCTION

Food is a source of micronutrients and macronutrients, which provides energy and functional molecules in relation to the body’s requirement. Food also contains numerous bioactive compounds, which can reduce or prevent the risk of various diseases [1]. Several bioactive compounds are found in plants such as ascorbic acid, carotenoids, anthocyanins, tocopherols, and polyphenols [2]. However, in animals, amino acid derivatives and non-protein nitrogen compounds are present, including, taurine, carnosine, coenzyme Q10, anserine, betaine, and creatine [3,4].

Recently, increased attention has been focused upon the nutritional and functional benefits of consuming meat, an excellent source of protein containing numerous beneficial compounds. Several previous studies have determined the antioxidant capacities of different meats, including, chicken, pork, beef, and fish [5]. It has been shown that chicken meat has the highest antioxidant capacity due to being rich in histidyl dipeptides, such as carnosine and anserine, which have significant antioxidant properties [6]. Jayasena and colleagues have also shown that differences in histidyl dipeptide content also exists between chicken genotypes, with Korean native chickens, especially in the chicken breast, having a higher histidyl dipeptide content compared with in commercial broiler [3].

The chicken meat industry in Thailand is growing due to the increase demand of both domestic and overseas markets [7]. Broiler is the main genotype in the Thai poultry industry because of the high growth performance resulting in low costs of production. However, there are other genotypes commercially produced for alternative meat consumption in Thailand, such as the black-boned and Thai indigenous chicken. The black-boned chicken has a special appearance but a high cost, although it is believed that their beneficial health effects lead to consumers paying more attention compared with other breeds of chickens [8]. Alternatively, Thai indigenous chickens, which are more expensive, have a slow growth rate, making it a popular meat for cooking due to its unique texture, good taste and low fat content [9].

To date, while most studies on chicken meat in Thailand have been focused on the growth performance, carcass and meat quality, there is limited data on the antioxidant capacity of chickens. Therefore, it remains unknown whether chicken meat obtained from different genotypes have different nutritional value and functional properties. Therefore, in order to provide health information of functional chicken meat, this study aimed to compare the macronutrients, antioxidant capacity, and bioactive antioxidants, including carnosine and anserine, of black-boned, Thai indigenous, and broiler breasts.

## MATERIALS AND METHODS

### Sample preparation

There are three genotypes in this study including Thai indigenous chicken, black-boned chicken, and broiler. Ten chickens of each genotype obtained from a commercial farm at Chiang Mai, Thailand were studied. Animal experimental designs were approved by the Animal Ethics Committee of the Faculty of Medicine, Chiang Mai University (No. 36/2562). All chickens were reared in one flock of a farm under identical conditions and were fed with formulated diets according to the genetic requirements. Sixteen weeks-old Thai indigenous chickens (weight 1.3 to 1.4 kg) and 20 weeks-old black-boned chickens (weight 1.2 to 1.3 kg) were used. Six weeks-old broilers (weight 2.1 to 2.3 kg) were a control group. They were slaughtered using the standard method and were chilled at 4°C for 24 h. After that, the breast meat without fat was immediately separated from carcass and was cut into small pieces. These small pieces were ground with a meat grinder. The minced breasts were packed in a vacuum bag and stored at −20°C before analysis.

### Proximate composition

All minced breasts were evaluated for their moisture, fat, protein and ash contents according to the AOAC [10]. Briefly, the moisture content was determined by oven-drying at 100°C for 24 h. Total protein (N×6.25) content was measured by the Kjeldahl method and fat content was determined using the Soxhlet extraction system. Ash content was measured by heating the sample in a furnace at 600°C for 6 h. The values are expressed as % (wet weight basis).

### Analysis of meat protein patterns

The molecular weight of proteins in the chicken breasts were determined by sodium dodecyl sulfate polyacrylamide gel electrophoresis (SDS-PAGE). The chicken breast was prepared according to Laemmli method using a slight modification [11]. The protein was extracted using the mixture of 8 M urea, 4% sodium dodecyl sulfate, and 4% 2-mercaptoethanol. A separating gel and a stacking gel containing 7.5% and 4.75% acrylamide, respectively, were used. The Full-Range Rainbow Molecular Weight Markers (17 to 230 kDa) purchased from BioDynamics Laboratory Inc (Tokyo, Japan) was used as the molecular weight standard. After gel was run at a constant voltage of 200 V, for 35 min, the gel was stained with coomassie brilliant blue G-250 for 1 h and then destained in a mixture containing 10% acetic acid and 40% methanol until protein bands were clearly visible. The intensity of each protein band was measured by Image J version 1.52a.

### Measurement of carnosine and anserine contents

The amount of carnosine and anserine was determined according to the modified method described by Mora and his group [12]. Chicken breast was homogenized with 0.01 N HCl. The supernatant obtained from centrifugation at 10,000 *g* for 20 min was mixed with acetonitrile and kept at 4°C for 20 min. Next, the mixture was further centrifuged for 10 min to provide supernatant for analysis using high performance liquid chromatography (HPLC). Twenty microliters of each sample were injected to a HPLC system with the Atlantis HILIC silica column (4.6×150 mm, 3 μm) as stationary phase. The mobile phase A contained 25 parts of 0.65 mM ammonium acetate, pH 5.5 and 75 parts of acetonitrile, while the phase B was composed of 70 parts of 4.55 mM ammonium acetate, pH 5.5 and 30 parts of acetonitrile mix. The flow rate was 1 mL/min for 16 min with a linear gradient (0% to 100%) from solvent A to B. A diode array detector was used at 214 nm to measure carnosine and anserine contents. The standard carnosine and anserine were obtained from Sigma Co. (St. Louis, MO, USA).

### Determination of antioxidant capacities

#### 2,2-Diphenyl-1-picrylhydrazyl assay

The free radical scavenging activities of chicken breasts were estimated according to the modified method of Jang et al [13]. Briefly, the supernatant of chicken breast was dissolved in fresh 0.2 mM 2,2-diphenyl-1-picrylhydrazyl (DPPH) solution and kept in a dark room for 30 min. The optical density of the mixture was measured at wavelength 517 nm. Trolox was used as a standard, while L-glutathione was a positive control. The percentage inhibition of sample was calculated by comparing with a solvent control and plotted against various concentration of samples.

#### 2,2′-Azino-bis 3-ethylbenzthiazoline-6-sulphonic acid assay

2,2′-Azino-bis 3-ethylbenzthiazoline-6-sulphonic acid (ABTS) radical scavenging activities were performed according to Re et al [14] with a slight modification. After dark incubation of the sample and fresh ABTS solution for 30 min, the reaction mixture was determined at wavelength 734 nm. The standard and positive control solutions and calculating method were used similar to above.

#### Ferric reducing antioxidant power assay

The ability of specimen to reduce ferrous ions was assessed according to the method of Benzie and Strain [15] with a slight modification. The ferric reducing antioxidant power (FRAP) solution containing 10 mM 2,4,6-tripyridyl-s-triazine and 20 mM ferric chloride in 300 mM sodium acetate buffer (pH 3.6), at a ratio of 1:1:10 (v:v:v), was added to a test specimen and incubated at 37°C for 30 min. The absorbance of resulting solution was estimated at 593 nm. L-glutathione positive control was used. The FRAP value of each sample was calculated from a Trolox standard curve and expressed as μM Trolox.

### Statistical analysis

A completely randomized design was applied throughout the experiments. The results were analyzed using one-way analysis of variance and mean comparisons were conducted using Duncan’s multiple range test. All statistical analysis was performed using Statistical Analysis System (SAS) Software (version 9.0; SAS Institute, Cary, NC, USA). The α level of significance was accepted at p<0.05.

## RESULTS AND DISCUSSION

### Proximate composition in breast of various chicken genotypes

The macronutrient contents in the chicken breasts of the black-boned and Thai indigenous genotypes were different (p<0.05) than that of the broiler (Table 1). The percentages of protein were not significantly different (p>0.05), yet their protein contents were much greater than that of the broiler (p<0.05). The broiler breasts contained a higher fat percentage compared with both the black-boned and Thai indigenous chickens (p<0.05). Notably, the percentages of moisture and ash were not different between each genotype (p>0.05).

The protein and fat content in chicken breasts are influ enced by several factors, with genotype playing a crucial role on the deposits of protein and fat in muscle [16]. We found that the fast-growing characteristic of broiler led to a lower protein but higher fat content compared with the slower growing characteristics of black-boned and Thai indigenous genotypes. Our results are in agreement with Jayasena and colleagues [17] who demonstrated that indigenous chickens, or crossbred chickens, had a lower percentage of fat compared to commercial breed chickens, which was unique character in these breeds. At the same live weight during harvesting, the slow-growing type was older than the fast-growing chicken [18]. This age difference may have affected muscle composition due to the protein turnover rate. Wattanachant and colleagues [19] reported that the protein percentage of Thai indigenous chickens increased from 21.5% to 24%, and moisture decreased from 77.8% to 71.6% at the age of 6 to 24 weeks, respectively. Typically, the amount of protein and fat in chicken muscle also depended on the feed quality and management [20]. The feeding of a diet containing soybean and palm oils were related to an increased fat composition in the broiler [21]. Corzo and his group, reported that protein accumulation in chickens were caused by a high amount of crude proteins and amino acids and a low amount of fat in the diets [22]. However, all genotypes in this study were fed with a similar formula diet. In addition, the macronutrient profile of the chicken meat showed that the breasts contained high protein but low fat when compared with pork and beef [5]. Therefore, slow-growing type chickens, including black-boned and Thai indigenous genotypes, might be an excellent source of macronutrients in the diet.

### Molecular size of chicken protein by gel electrophoresis

**Figure 1:**
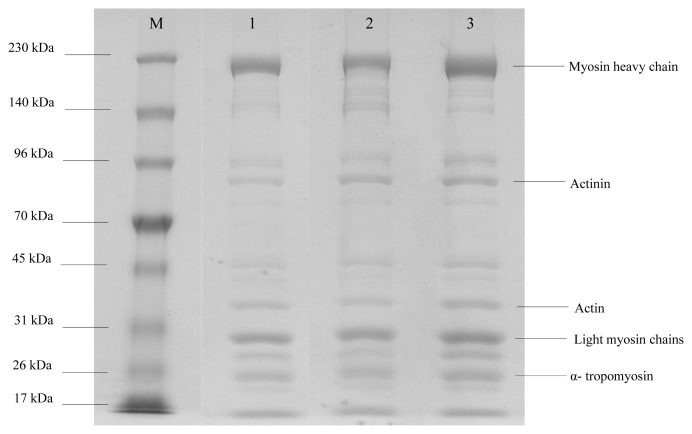
Protein pattern of chicken breast muscle. Sodium dodecyl sulfate-polyacrylamide gel electrophoresis was carried out with a vertical system using 7.5% and 4.5% acrylamide for separating and stacking gels, respectively. Ten μL of 1 mg/mL of protein sample was loaded in each lane. Gel was stained with Coomassie blue R-250 after protein separation. Lane M, standard protein marker; Lane 1, broiler; Lane 2, black-boned chicken; Lane 3, Thai indigenous chicken.

**Figure 2:**
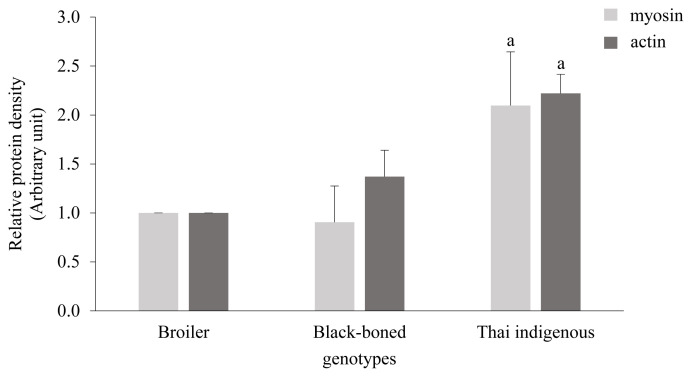
Effect of chicken genotypes on myosin and actin contents in breast muscle. Data are presented as mean±standard error. ^a^ Significant difference from broiler (p<0.05).

The patterns of chicken breast proteins in all genotypes are shown in Figure 1. It was found that chicken proteins had bands in molecular weight ranging from 17 to 230 kDa, with the protein pattern in each genotype not different. The myosin heavy chain was the predominant band of chicken protein detected by SDS-PAGE. Actinin, actin, light myosin chains, and α-tropomyosin were also found. Myosin and actin are the main proteins found in animal muscle and are the principal proteins which provide several peptides that are associated with beneficial properties, such as antioxidant and anti-inflammatory activity [23–25]. Thai indigenous chickens were found to have the most abundant myosin and actin contents while the broiler had the least amount of these proteins (Figure 2).

The differences between the amount of myosin and actin contents may be related to the different chicken genotypes. Liu and colleagues have shown that the individual genetic pattern can influence the degradation of muscle fiber type during the postmortem aging period and affect meat quality [26]. Moreover, the protein bands of the broiler were of lower intensity than the Thai indigenous genotype. Together, our findings may therefore suggest that the diverse protein content may have been caused by chicken ageing. These results suggest that Thai indigenous chickens are a valuable source of bioactive peptides.

### Carnosine and anserine contents

**Figure 3:**
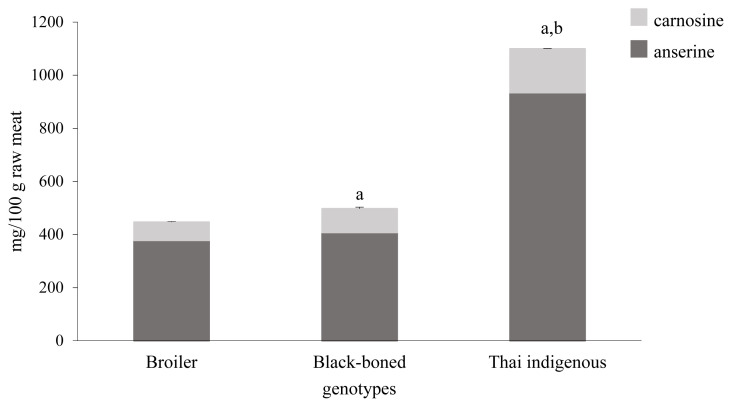
Effect of chicken genotypes on total carnosine and anserine contents in breast muscle. Total amount of carnosine and anserine was measured by high performance liquid chromatography and presented as mg per 100 g in raw meat. ^a^ Significant difference from broiler (p<0.05). ^b^ Significant difference from black-boned chicken (p<0.05).

The carnosine and anserine contents within the different chicken breast genotype are shown in Figure 3. The majority of breast muscle consisted of muscle fiber type IIB, the fast twitch glycolytic type with low myoglobin, which produces ATP molecules under anaerobic metabolism leading to lactic acid and pH imbalance in this area [9,27]. It has been reported that carnosine and anserine facilitates pH regulation in the body, thereby playing an important role in maintaining the balance of anaerobic glycolysis [28]. The Thai indigenous chickens contained the highest amount of carnosine and anserine followed by the black-boned and broiler genotypes, respectively. These findings are consistent with Peiretti and his group, who reported that the anserine content was greater than that of the carnosine contents [29]. Our study showed that the amount of anserine in the Thai indigenous genotype was 2.3- and 2.5-fold higher than in the black-boned and broiler, respectively. Anserine, a histidine peptide, is an N-methylated derivative of carnosine mostly found in poultry meat, whereas carnosine exists mainly in beef and pork [30]. The anserine and carnosine ratio of pork and beef was less than 0.2. The ratio of anserine and carnosine content in breast of Thai indigenous, black-boned and broiler was 5.6, 4.5, and 5.4, respectively, which were in line with previous findings [29].

The amount of carnosine and anserine is related to type of muscle fiber, genotype, sex, age, and breeding [6,31]. Although, the age of the chickens used in our study typically varies according to market demand, this factor did not influence carnosine and anserine content in native Korean chickens [32]. Moreover, Jaturasitha and colleagues reported that the content of muscle fiber type IIB were not significantly different (p>0.05) between fast-growing and slow-growing types [9]. However, carnosine and anserine contents in slow-growing genetic chickens, including black-boned and Thai indigenous chickens, were different. Meat type might be categorized by color depending on the myoglobin content [33]. Red meat is rich in the oxygen-carrying pigment, myoglobin, while white meat contains less myoglobin resulting in a different anaerobic glycolytic rate. This may provide an explanation why black-boned chickens have a low carnosine and anserine content. Furthermore, breeding may affect the expression of the enzymes and transporters involved in carnosine and anserine metabolism. Intarapichet and Maikhunthod [34] suggested that the carnosine content in native chickens is significantly different from that found in crossbred chickens.

### Antioxidant capacities

The antioxidant capacity of chicken breasts in all genotypes using 3 *in vitro* antioxidant tests are shown in Table 2. Each 1.25 mg chicken breast containing 289 to 313 μg of protein presented a varied range of antioxidant capacities measured via DPPH assay. The Thai indigenous chicken had the highest antioxidant capacity followed by the black-boned chicken and the broiler. However, the broilers exhibited the strongest electron donating capacity using the ABTS assay at 0.75 mg of chicken breast. Furthermore, the FRAP value of the Thai indigenous chicken had the greatest value (1.80) followed by the black-boned chicken (1.64) and the broiler (1.35) at 0.20 mg. The antioxidant results of each chicken breast were obtained from the DPPH test, which determined that the proton-donating capacity was consistent with the FRAP method, which measured ferric ion-reducing capacity. These results were in line with Serpen and his group that showed that the antioxidant activity obtained from an ABTS test contrasts with DPPH and FRAP assays. Moreover, a higher antioxidant value in broiler breast, evaluated by the ABTS method appropriated to both polar and non-polar compounds, may be due to a high fat content. However, DPPH radical is likely more selective than ABTS radical in the reaction with proton donors [35]. In addition, our study found that antioxidant capacities detected by DPPH and FRAP assays were correlated to the content of carnosine and anserine in chicken breast.

## CONCLUSION

The antioxidant capacities and macronutrient and bioactive compound contents of chicken breasts were greatly influenced by genotype. The breast obtained from Thai indigenous chickens contained low fat, enriched protein, and high bioactive compounds, which may have health benefits compared with other commercial genotypes. However, other chicken portions, which are popular in some regions, should be further investigated for their functional compounds.

## Figures and Tables

**Table 1:** Macronutrients of breast muscle from broiler, black-boned chicken, and Thai indigenous chicken

Genotypes	Protein (%)	Fat (%)	Moisture (%)	Ash (%)
Broiler	23.1±0.16	3.07±0.34	72.6±0.10	1.13±0.02
Black-boned	24.4±0.17^a^	1.23±0.09^a^	72.4±0.16	1.07±0.02
Thai indigenous	25.3±0.69^a^	0.50±0.05^a^,^b^	72.2±0.02	1.17±0.02

Data are presented as mean±standard error of mean.

a Significant difference from broiler (p<0.05).

b Significant difference from black-boned chicken (p<0.05).

**Table 2:** Antioxidant capacities of breast muscle from broiler, black-boned chicken, and Thai indigenous chicken

Genotypes	% Inhibition		FRAP value (μM Trolox/g sample)

	DPPH assay	ABTS assay	
Broiler	11.4±0.45	19.8±1.33	1.35±0.10
Black-boned	19.1±0.31^a^	16.8±0.53^a^	1.64±0.11
Thai indigenous	27.2±0.19^a^,^b^	14.7±0.74^a^	1.80±0.15^a^

Data are presented as mean±standard error of sample mean.

DPPH, 2,2-diphenyl-1-picrylhydrazyl; ABTS, 2,2′-azino-bis 3-ethylbenzthiazoline-6-sulphonic acid; FRAP, ferric reducing antioxidant power; GSH, reduced glutathione.

a Significant difference from broiler (p<0.05).

b Significant difference from black-boned chicken (p<.05).
